#  Sudden Intrauterine Unexpected Fetal Death Syndrome and Sudden Infant Death Syndrome 

**Published:** 2014-08-15

**Authors:** Teresa Pusiol, Doriana Morichetti, Maria Grazia Zorzi, Luigi Matturri, Anna Maria Lavezzi

**Affiliations:** 1 Institute of Anatomic Pathology, Rovereto Hospital, Rovereto, Trento; 2 “Lino Rossi” Research Center of the Milan University, Italy

**Keywords:** SIDS, SIUDS, Autonomic Nervous System, Neuropathology, Environmental Risk Factors

**Fig.1:**
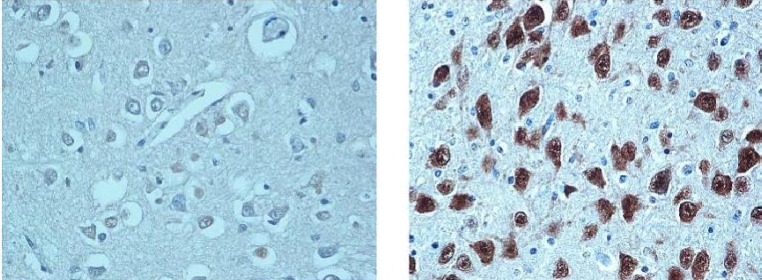
**Left**. NeuN-immunonegative neurons in a pontine histological section of a SIDS victim dead at 2 months. **Right.** NeuN_immunopositivity in an equivalent section of a control case (late fetal death -38 gestational weeks) for comparison - NeuN immunostaining. Magnification: 40×

On 26th of April 2012, the “Autonomous Province of Trento” has brought into effect the Law n.31/2006 “Regulations for Diagnostic Post Mortem Investigation in Victims of the Sudden Infant Death Syndrome (SIDS) and Unexpected Fetal Death”, establishing an agreement with the “Lino Rossi” Research Center of the University of Milan. The Law states that all infants suddenly died within the first year of life, suspected of SIDS, and all fetuses died after the 25th week of gestation without any apparent cause, must undergo an anatomopathological examination^[^^1^^]^. We report the results until now obtained, focusing on the neuropathology of reflexogenic sudden perinatal and infant death. The study included 9 Sudden Intrauterine Unexpected Fetal Death Syndrome (SIUDS) (aged 19-40 gestational weeks) and 3 SIDS (aged from 20 hours to 6 months), occurring in the Autonomous Province of Trento (North Italy) in a 2-year period (2011-2012). For each case, all available information about pregnancy, fetal development and delivery and, in cases of infant death, about the environmental and familiar situation where the death occurred, besides information related to the potential risk factors (such as maternal smoking, maternal obesity, feeding, position the baby was last left in), were collected and categorised during post-mortem family interviews. The information sheets were recorded in a dedicated data bank, according to the art. 3 of the Law 31/2006, predisposed by the Autonomous Province of Trento and including two subsections: one for fetal loss and another for infant deaths. After removal two fresh samples of cerebral cortex (0.5-1 cm^3 ^thick) for the genetic and toxicological investigations, respectively, the brainstem and the cerebellum, where the main vital centers are located (cardiorespiratory, arousal, upper digestive tract, etc.), were processed and embedded in paraffin. Transverse serial sections were made at intervals of 30 μm. For each level, six to seven 5 μm sections were obtained, two of which were routinely stained for histological examination using alternately hematoxylin-eosin and Klüver Barrera stains. The other two sections were employed for immunohistochemical detection of the neuronal nuclear antigen 3 (NeuN). The remaining sections were saved for further investigations and stained as deemed necessary. The routine histological evaluation of the brainstem was focused on the locus coeruleus, parafacial/facial complex, superior olivary complex, retrotrapezoid nucleus, superior olivary nucleus, parabrachial/Kölliker-Fuse complex in the pons/mesencephalon; on the hypoglossus, the dorsal motor vagal, the tractus solitarius, the ambiguus, the pre-Bötzinger, the inferior olivary and the arcuate nuclei in the medulla oblongata and the intermediolateral nucleus in the spinal cord. We can summarize the results as follows: 1) Both unexplained fetal and infant deaths share common congenital anomalies of the central autonomic nervous system, so indicating that SIUDS should not be regarded as distinct from SIDS. 2) Noteworthy in the brainstem of the unexplained fetal death is the frequent association of the hypoplasia of the facial/parafacial complex with the hypoplasia of the raphe system. In particular, the hypodevelopment of the parafacial nucleus, consisting of “pre-inspiratory” neurons with the main function of hierarchical modulation of the breathing circuitry, is a very frequent finding in unexplained stillbirths. The raphe neurons in fact, known as major producers of serotonin and responsible for the serotonergic transmission during intrauterine life, play a trophic role in the neuronal development of fetal brains. A dysfunction in serotonergic transmission during intrauterine life could affect all neuronal structures checking vital functions, thus preventing the fetal life. 3) The NeuN protein is not expressed in both SIUDS and SIDS cases (Fig. 1). These congenital anomalies are likely to disrupt the vital actions by reflex mechanism, centered within bulbospinal structures in the domain of baro-(mechano)-chemoreflexes. These respond to variations in blood pressure and chemical breathing-depended components of blood and cerebrospinal fluid (e.g. pO2, pCO2, pH).

The pathological involvement of the vagal-glossopharingeal neuronal circuitry explains some lethal reflexogenic mechanism, quoted in the literature as “drive”, “death-feigning” and/or “Ondina’s course” reflexes. Moreover, parahypoglossal glossopharingeal brainstem damage can promote suffocation by tongue hypotonia during supine sleep, especially if an abnormal reticular formation 4 impairs respiration and/or gastro-esophageal reflux goes into trachea. Overall the results obtained in the 12 cases of sudden death reported here, in agreement with those highlighted in our previous study^[^^2^^]^ performed on a very large survey of perinatal deaths (140 SIDS, 95 SIUDS and 78 controls), show the common congenital origin of SIUDS and SIDS and support the statement of the National Institute of Child Health and Human Development: “SIDS is a developmental disorder. It takes its origin from fetal development”. In the world, only Italy has a national law that orders “Regulations for Diagnostic Post Mortem Investigation in Victims of the SIDS and Unexpected Fetal Death”. This law can be an example for the other nations in the world, and we believe it should be known. The present paper is the first result of the application of the law. The province of Trento (North Italy) applies this law because it is an autonomous province. The use of NeuN immunohistochemistry is another new important marker in the study of this pathology. 
